# Analysis of Diurnal Variations in Heart Rate: Potential Applications for Chronobiology and Cardiovascular Medicine

**DOI:** 10.3389/fphys.2022.835198

**Published:** 2022-03-08

**Authors:** Tao Zhang, Xiaojiao Du, Yue Gu, Yingying Dong, Wei Zhang, Zhirong Yuan, Xingmei Huang, Cao Zou, Yafeng Zhou, Zhiwei Liu, Hui Tao, Ling Yang, Gang Wu, John B. Hogenesch, Chengji J. Zhou, Fei Zhou, Ying Xu

**Affiliations:** ^1^Cambridge-Su Genomic Resource Center, Jiangsu Key Laboratory of Neuropsychiatric Diseases Research, Suzhou Medical College of Soochow University, Suzhou, China; ^2^Division of Cardiology, The First Affiliated Hospital of Soochow University, Suzhou, China; ^3^School of Mathematical Sciences, Soochow University, Suzhou, China; ^4^Division of Cardiology, Suzhou Dushu Lake Hospital of Soochow University, Suzhou, China; ^5^Divisions of Human Genetics and Immunobiology, Center for Chronobiology, Department of Pediatrics, Cincinnati Children’s Hospital Medical Center, Cincinnati, OH, United States; ^6^Department of Biochemistry and Molecular Medicine, School of Medicine, University of California, Davis, Sacramento, CA, United States

**Keywords:** heart rate, diurnal patterns, biomarker, Holter monitor, cardiovascular diseases

## Abstract

Circadian factors likely influence the occurrence, development, therapy, and prognosis of cardiovascular diseases (CVDs). To determine the association between the heart rate (HR) diurnal parameters and CVD risks, we designed an analytical strategy to detect diurnal rhythms of HR using longitudinal data collected by clinically used Holter monitors and wearable devices. By combining in-house developed algorithms with existing analytical tools, we obtained trough phase and nocturnal variation in HR for different purposes. The analytical strategy is robust and also sensitive enough to identify variations in HR rhythms influenced by multiple effectors such as jet lag, geological location and altitude, and age from total 211 volunteers. A total of 10,094 sets of 24-h Holter ECG data were analyzed by stepwise partial correlation to determine the critical points of HR trough phase and nocturnal variation. The following HR diurnal patterns correlate with high CVD risk: arrhythmic pattern, anti-phase pattern, rhythmic patterns with trough phase less than 0 (extremely advanced diurnal pattern) or more than 5 (extremely delayed diurnal pattern), and nocturnal variation less than 2.75 (extremely low) or more than 26 (extremely high). In addition, HR trough phases from wearable devices were nearly identical to those from 24-h Holter monitoring from 12 volunteers by linear correlation and Bland-Altman analysis. Our analytical system provides useful information to identify functional diurnal patterns and parameters by monitoring personalized, HR-based diurnal changes. These findings have important implications for understanding how a regular heart diurnal pattern benefits cardiac function and raising the possibility of non-pharmacological intervention against circadian related CVDs. With the rapid expansion of wearable devices, public cardiovascular health can be promoted if the analytical strategy is widely applied.

## Introduction

Human physiological and pathological parameters are tightly controlled by circadian rhythms (Zhang and Kay, 2010; López-Otín and Kroemer, 2021). Diurnal variations in physiological status (e.g., heart rate (HR) and blood pressure) are commonly observed in the human cardiovascular system. Chronic disruption of circadian rhythms, such as with shift work, jet lag, social interaction, feeding patterns, or malfunction of the circadian clock, can lead to the development of cardiovascular disease (CVD) [reviewed in (Guo and Stein, 2003; Takeda and Maemura, 2011; Thosar et al., 2018; Crnko et al., 2019)]. The incidence of CVD also varies within a 24-h period (Waters et al., 1984; Jeyaraj et al., 2012) due to the raising and lowering of HR and blood pressure, early morning alterations in autonomic nervous system activity, and circadian production of hormones such as cortisol (Oster et al., 2006). Therefore, identifying the association between circadian rhythmicity, especially the rhythmicity of the cardiovascular system, and CVD could be valuable for CVD risk prediction, diagnostic assays, and improved treatment.

A number of measurements, such as morningness-eveningness questionnaires (Horne and Östberg, 1976) and dim-light melatonin onset (DLMO) (Lewy et al., 1999), have been used to estimate human circadian time, which was further defined as chronotypes (Adan and Almirall, 1991; Roenneberg et al., 2003). After a pioneering study involving transcriptomic profiling to infer the human circadian phase, various methods have been developed to establish robust and accurate human circadian biomarkers (Ueda et al., 2004; Hughey, 2017; Braun et al., 2018; Ruben et al., 2018; Wittenbrink et al., 2018; Wu et al., 2018). However, none of these methods directly reflect circadian rhythmicity in the cardiac system. Twenty-four-hour Holter monitoring offers continuous and accurate HR recordings, but it is somewhat inconvenient to use. Notably, the recent explosive increase in wearable healthcare devices has provided a simpler method for health monitoring including heart rate (Lu et al., 2020). The long-term HR tracking enabled by wearable biometric devices (wristbands) also generates a sufficient amount of data for circadian analyses of the cardiac system. Furthermore, accuracy of wearable healthcare devices is well estimated and datasets recorded by these devices were analyzed to characterize the relationship between HR diurnal patterns and health problems (Haghayegh et al., 2019; Zhang et al., 2019; Kalanadhabhatta et al., 2021).

Therefore, we developed an analytical strategy to dissect the HR diurnal parameters. Using the strategy, we tried to address the association of HR diurnal rhythmicity changes with the risk of CVD based on population-level clinical Holter data and evaluate the accuracy of HR diurnal parameters from wristband-data.

## Materials and Methods

### Clinical ECG Data

A total of 10,094 historical Holter data sets were collected from the Division of Cardiology, the First Affiliated Hospital of Soochow University, covering the corresponding patients from Sep 2010 to Jul 2014. Holter data sets were then screened for data integrity and to exclude patients with artificial pacemakers for an unbiased analysis of diurnal pattern. A total of 9,922 data sets were subjected to further analyses. Simplified HR data at 1-minute frequency as well as diagnostic conclusions were exported using a customized module of the Holter software, ECG Lab (Biomedical Instruments, Shenzhen, China). This retrospective study design was approved by the Ethics Review Board of the hospital (Application ID: 2019025).

### Cardiovascular Diseases Indices

Holter data sets containing diagnostic conclusions were given by experienced cardiac physicians based on clinical guidelines (AHA/ACCF/HRS Recommendations for the standardization and Interpretation of the Electrocardiogram). Cardiovascular-related pathological indicators noted in the diagnostic conclusions of the Holter data were defined as CVD indices for correlation analyses. All indices were extracted using a Perl script and classified into seven categories with 13 subgroups (Supplementary Table 1).

### Dim-Light Melatonin Onset

Twelve volunteers were gathered in two lightproof rooms from 18:00 to 24:00. Saliva samples were collected every 30 min, stored in a refrigerator, and analyzed using an ELISA kit (IBL International, Switzerland). The melatonin onset for each individual was determined by the hockey stick method in MATLAB (Danilenko et al., 2014).

### Volunteers

The Morningness-eveningness questionnaire (MEQ) was distributed online *via* a social network (Liu et al., 2020). Questionnaire participants were then invited to join the wristband-based study. Between November 2017 and September 2019, 211 volunteers (72 males, 139 females) were recruited to wear a smart wristband for at least 1 month. The investigation conforms with the principles outlined in the *Declaration of Helsinki* and was approved by the Ethics Committee of Soochow University (ECSU-201800098).

### Wristband Data Collection and Analysis

The wearable devices were purchased from two independent vendors, Fitbit and Huawei. HR were continuously recorded by wristband for at least one week between November 2017 and September 2019. All the data are only used for research purpose. All volunteers provided an electronic informed consent for their data usage when they first registered and initialized their devices. Data in the wristband are transferred to the vendor cloud server by smartphone app. Mobile phone application and the vendor data server also provide sleep stages in the app: awakening, light, deep, and REM sleep. Then we synchronized these data to our local server by API docking and performed further analysis. Night-Day ratio, rhythmicity and critical points of HR data from wristbands were identified using R. Diurnal parameters, such as trough phase and nocturnal variation were estimated by “The Key analytical algorithm for HR data” in MATLAB (Supplementary Material). All the diurnal parameters of HR are described in Supplementary Table 2.

### Analytical Algorithm for Heart Rate Data

To avoid interference from daytime activity, nighttime HR data were searched and selected for analyses. In brief, the key analytical algorithm for HR data was developed based on Holter data using exported simplified HR data and then tested with wristband data and synthetic data to verify its feasibility. We first filtered the data with a Butterworth filter for denoising. Then, we used the sliding window approach to automatically distinguish rest and active periods by labeling the HR falling and rising slopes instead of fixed nighttime and daytime periods. By comparing these data with the threshold calculated from the filtered HR data, the first time point of the sliding window for which all HRs were below the threshold was considered the starting point of the night period. A similar treatment was performed to determine the end of the night. Next, the least squares method was used to perform cosine fitting with nighttime HR data. The lowest fit HR value and the trough phase time were automatically identified. Oscillations with period less than 1 h are removed to exclude the influence of sleep cycles and other high frequency fluctuation. The HR nocturnal and diurnal variations during the nighttime and the whole day/night cycle of each subject were calculated using the daily average HR, the mean resting HR and the lowest fit HR. Detailed descriptions of the algorithm are included in Supplementary Material.

### Synthetic “Heart Rate Data” Generation

To verify reliability, we conducted a test using synthetic data sets. We defined a periodic signal consisting of the trough of the sine curve (night time) and peaks of three bell-shaped curves (day time) with a known period (24 h) and phase. A standard normal random perturbation was used to simulate noise. The script was repeated 1,000 times to generate the synthetic data with values of phase chosen uniformly at random from 2 p.m. to 2 p.m. on the next day. A standard normal random perturbation was used to simulate measurement error and experimental noise. Our algorithm was then applied to the data collection for analyses.

### Jonckheere-Terpstra-Kendall Rhythmicity Analysis (JTK_CYCLE)

To analyze the rhythmicity of the 24-h heart rate of each sample, JTK_CYCLEv3.1 was used to process hourly binned HR data. Then, the ratio of the average nighttime HR to daytime HR (N/D) was calculated to determine the in-phase or antiphase. For all data sets, permutation-based *P*-values (ADJ.P) were applied. JTK_CYCLE analyses with a two-sided *P*-value less than 0.05 were considered statistically significant (Hughes et al., 2010).

### Heatmap

To better illustrate the amplitude and phase patterns of daily heart rate, each HR time series was normalized on a [0,1] scale. A heatmap was plotted using “pheatmap” in the R package (R version 3.6.1). In the rhythmic cluster, HR time series were ordered according to trough phases.

### Critical Point Determination

Considering that two key parameters for the HR diurnal rhythm, trough phase and nocturnal variation, are continuously distributed in the population, we built a model with R language to perform stepwise partial correlation analyses. Stepwise partial correlation analyses for CVD indices were carried out with gradually narrowed parameters from either direction. A typical value level for step size was 0.01 to obtain enough subjects, while age and sex were adjusted with the R package “ppcor.” Range boundaries with a correlation coefficient greater than 0.1 and a significant *P* value were considered to indicate the critical point beyond which individuals were considered to have a significantly higher risk of CVD. Partial correlation analyses with a two-sided *P* value less than 0.05 were considered statistically significant.

### Statistics

Linear regression was used to evaluate continuous relationships between the HR trough phase from Holter and wristband data. Linear regressions were also used to evaluate continuous relationships between HR trough phase from wristband data and MEQ scores and between HR trough phase from wristband data and predicted DLMO. Partial correlation analyses were conducted to determine the correlation between HR diurnal parameters and CVD indices. Using GraphPad Prism 8, one-way ANOVA with Bonferroni *post hoc* testing was used to evaluate the association between disease occurrence and HR diurnal patterns by inputting the baseline characteristics of CVD indices from ECG data and the following patterns: arrhythmic pattern, antiphase pattern and rhythmic pattern. All statistical analyses with a two-sided *P* value less than 0.05 were considered statistically significant.

## Results

### Analytical Strategy and Algorithm Development

**FIGURE 1:**
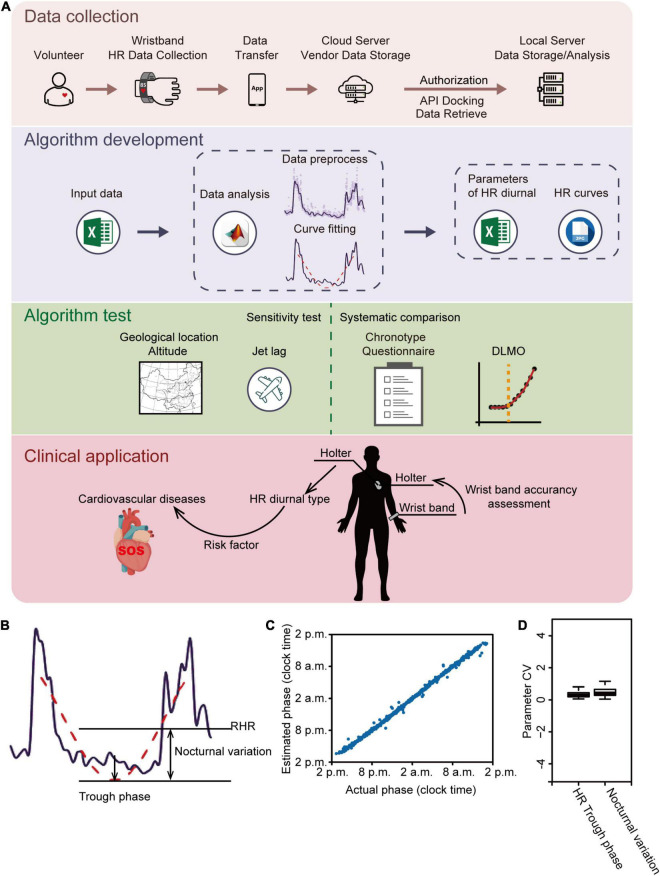
Analytical strategy and algorithm development. **(A)** Schematic illustration of the analytical strategy. Heart rate (HR) data from both volunteers and clinic patients were collected and subjected to analysis by an in-house developed algorithm. HR diurnal parameters were generated by the algorithm and further tested by analyses of geological locations and altitude; traveling across time zone; chronotype questionnaire comparison; and DLMO. Finally, HR diurnal parameters were used for CVD risk prediction. **(B)** Representative HR data showing the algorithm development. Purple line: filtered HR data; red dashed line: fitted HR curve during resting time; RHR: resting mean HR. **(C)** Algorithm reliably estimates trough phase of synthetic daily HR data. A test set of 1,000 daily HR data was generated with trough phases uniformly chosen from 2 p.m. to 2 p.m. on the next day and nocturnal variation ranging from 30 to 60. Estimated phase is plotted as a function of the actual phase showing a strong linear correlation (*r* = 0.997). **(D)** The interday coefficient of variation (CV) of HR trough phase and nocturnal variation was analyzed using data of the first three workdays retrieved from all 211 volunteers. The box plot shows the distribution of SD values for each parameter.

Our study includes the synergistic evaluation of HR data from volunteers and clinical patients by using the strategy illustrated in Figure 1A. HR data were subjected to analysis using an in-house developed algorithm consisting of data pre-processing and curve fitting analysis (Figure 1A). The diurnal parameters included trough phase which is the timing of the lowest point in the fitted curve, and nocturnal variation which is the difference between the resting average HR (RHR) and the fitted minimum HR (Figure 1B and Supplementary Figure 1A). To test the reliability and accuracy of our algorithm, synthetic “HR data” were generated with a variable trough phase. The result showed that the estimated trough phase was in agreement with the actual trough phase of synthetic “HR data” (Figure 1C), indicating that our algorithm is reliable. Furthermore, the inter-day coefficient of variances of the trough phase, nocturnal variation and other parameters were very low between three workdays for each of 211 participants, except for the falling onset time and the offset time (Figure 1D and Supplementary Figure 1B), which suggests that diurnal parameters were robust for each individual within a certain period. We thus excluded the falling onset/offset time as robust parameters, which are likely interfered by variations of sleep time and activity.

### Characterization of Heart Rate Diurnal Parameters Derived From Wearable Devices

**FIGURE 2:**
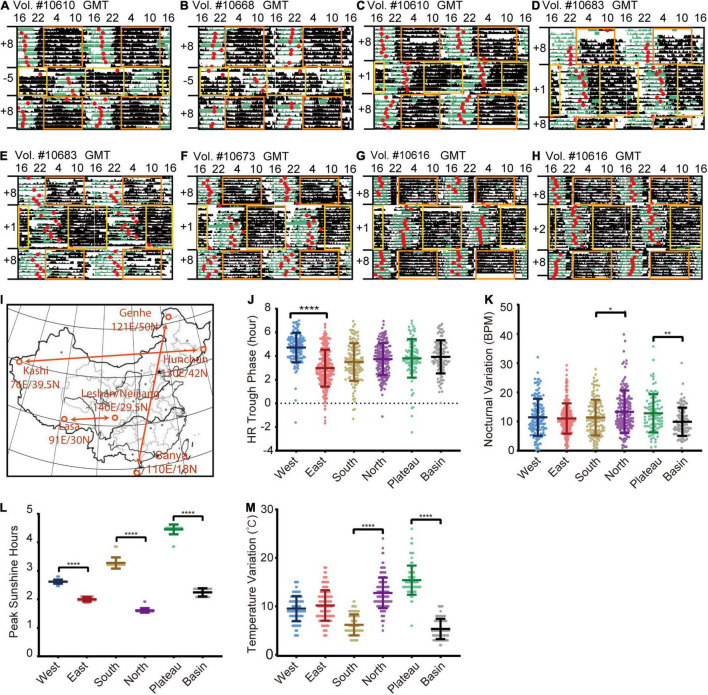
HR rhythmic changes in the different time zones. **(A–H)** Representative HR actograms of different volunteers who experienced jetlag of 12 h **(A,B)**, 7 h **(C–G)** and 6 h **(H)**. The period from an average low-level transition to a relatively high-level and then back to low-level was marked as black bar, and the time of the trough phase of HR (red dot) were well-aligned before traveling. The sleep scores generated by the wristband were labeled as green bars. After flying to a new time zone, the watch time is synchronized (delay or advance) with the local time, which will lead to overlapping or missing time, resulting in wristband data overwritten or loss. Blank regions are that the real-time data was overwritten when wristbands were synchronized to the new time zone. The yellow and orange rectangles represent daytime (8:00 to 20:00) in different time zones. **(I)** Illustration of geological locations selected for comparison. West: Kashi in Xinjiang province (76E/39.5N); East: Hunchun in Heilongjiang Province (130E/42N); North: Genhe in Inner Mongolia (121E/50N); South: Sanya in Hainan province (110E/18N); Plateau: Lasa in Tibet (91E/30N); Basin: Leshan and Neijiang in Sichuan province (∼104E/29.5N). HR data collected in an entire month (January) were used for comparison. **(J)** Quantification of HR trough phase values in different locations. **(K)** Quantification of nocturnal HR variation in different locations. **(L)** Quantification of peak sunlight hours in different locations. **(M)** Quantification of temperature variation in different variation. Daily results of individual volunteers are considered as single data points for quantification due to limited numbers of volunteers in different regions. The numbers of volunteers are: West: 12 volunteers (*N* = 140), East: 19 volunteers (*N* = 230), South: 16 volunteers (N = 136), North: 17 volunteers (*N* = 171), Plateau: 9 volunteers (*N* = 94), Basin: 12 volunteers (*N* = 107). Error bars indicate the mean ± SD *p < 0.05; **p < 0.01; ****p < 0.0001.

To test the sensitivity of the method, we evaluated the corresponding changes in responses to well-known two-step jet lag. As shown in Figures 2A,B, the HR trough phases (red dots) of volunteer #10610 and #10668 graphically displayed a similar entrainment to westward time-zone (12-h time difference), which did not completely correspond to the adjustment rate of sleep onset, while they had distinct re-entrainment in response to eastward time-zone transition (Figures 2A,B). Other volunteers’ HR trough phases can also be found to entrain to the new time zone (Figures 2C-H).

To further examine to what extent HR can reflect cardiovascular circadian changes in response to environmental cues, most importantly, to visible light and external temperature cues (Egg et al., 2013; Wu et al., 2017), we retrieved 85 wristband-based HR data from representative areas in January, 2019 (Figure 2I). The mean HR trough phase of 12 wristbands with a total of 140 daily measurements (age: 36.68 ± 9.6 years) from Kashi (76E/39.5N) was significantly delayed compared with that of 19 wristbands with a total of 230 daily measurements (age: 39.38 ± 8.6 years) from Hunchun (130E/42N) (Figure 2J). This difference corresponds to a delayed sunrise time in west regions in spite of the unified Beijing Time, which is consistent with previous reports (Roenneberg and Merrow, 2007; Roenneberg et al., 2007, 2013; Fischer and Lombardi, 2021). Interestingly, by comparing HR data of 17 wristbands with total 171 daily measurements (age: 39.27 ± 13.7 years) from Genhe (121E/50N) and 26 wristbands with 232 daily measurements (age: 36.32 ± 9.7 years) from Sanya (110E/18N), we found that the average nocturnal variation was higher among those in northern regions (Figure 2K). When we compared 9 wristbands with 94 daily measurements (age: 33 ± 8.5 years) from Lasa (Plateau, 3,684 meters above sea level, 91E/30N) with 21 wristbands with a total of 178 daily measurements (age: 32.78 ± 9.8 years) from Leshan and Neijiang (Basin, 500 meters above sea level, ∼104E/29.5N), unexpectedly, we found that the average nocturnal variation of Lasa individuals was higher than that of Sichuan individuals. Then we estimated two dominant environmental cues: the peak sunlight hours (irradiance of 1,000 w/m^2^) (Refinetti, 2019) and the daily temperature variation (delta temperature) in the above indicated area in January, 2019. As shown in Figure 2L, the duration of peak sunlight is not a direct factor for increased nocturnal variations in the Lasa or northern areas because it was long in Lasa and short in the northern and basin areas (Figure 2K). Furthermore, we observed significantly greater daily temperature variation in the Lasa and northern areas compared with the southern and basin areas, indicating a possible link between ambient temperature variation and cardiovascular circadian changes (Figure 2M). Altogether, our data here proves that the algorithm can track an individual’s circadian state, and heart diurnal paraments can reflect circadian entrainment.

### HR Diurnal Parameters Highly Correlate With Other Timing Parameters

**FIGURE 3:**
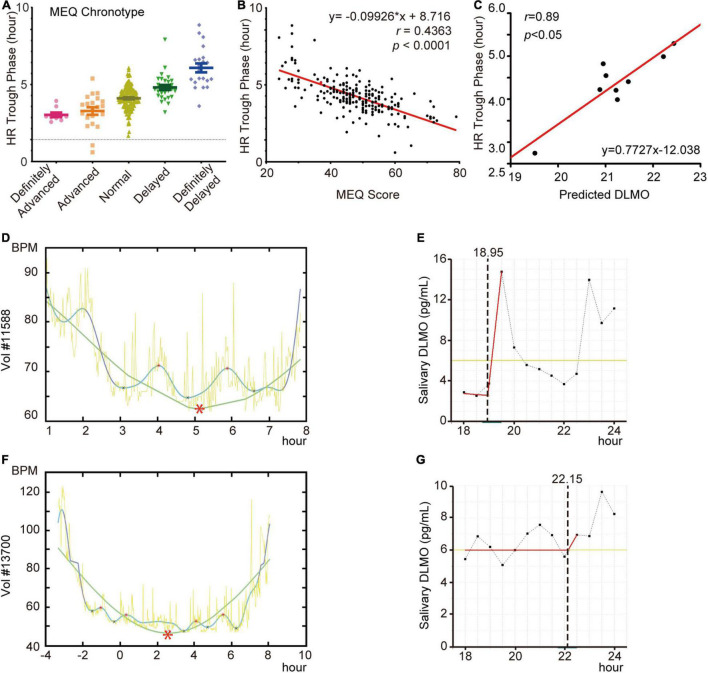
HR diurnal parameters highly correlate with other timing parameters. **(A,B)** Comparison of HR trough phase values and MEQ chronotypes **(A)** and scores **(B)** of volunteers (*N* = 211). **(C)** Linear regression of HR trough phase values and DLMO results from 9 volunteers. **(D)** HR curves of volunteer #11588. **(E)** DLMO prediction curves of volunteer #11588. **(F)** HR curves of volunteer #13700. **(G)** DLMO prediction curves of volunteer #13700. Red asterisks in HR curves indicate the trough. Dotted lines indicate predicted DLMO timepoints.

To address whether the circadian phase of HR can be evaluated by other circadian output markers, we first administered the chronotype questionnaire (MEQ) to 211 volunteers with wristbands and applied our in-house algorithm to their HR data. We found a strong correlation between the HR trough phase and MEQ chronotypes (Figure 3A) or MEQ scores (Figure 3B), but HR trough phases were not always concordant with the MEQ chronotypes or MEQ scores.

Next, to study the relationship between the HR trough phase and melatonin phase, salivary samples were collected from 11 participants and calculated their DLMO (Supplementary Figure 2) as described previously (Danilenko et al., 2014). Following exclusion of two outliers from the analysis, the agreement of HR trough phase with DLMO was significant and robust when estimated by linear regression (*r* = 0.89, *p* < 0.05) (Figure 3C). This suggested that most participants’ HR trough phases were aligned with the DLMO. We then considered the two apparent outliers, participants #11588 and #13700, which were excluded from the above analysis. When their DLMO and HR trough phases were further analyzed, the HR trough phase was approximately 05:10 (Figure 3D) and matched the MEQ score as a definitely delayed HR pattern. However, we observed that participant #11588 experienced an early surge in melatonin at approximately 19:30 (Figure 3E). This participant had a nap during this short time and was then unable to fall asleep until approximately 04:00. In addition, the peak of melatonin in participate #13700 was not detected, while the trough phase occurred at approximately 02:30 (Figures 3F,G). Altogether, our results showed that the phase of HR is unable to be evaluated using a simply MEQ or DLMO substitution, but there is a stable relationship between them as reported in previous work (Roenneberg and Merrow, 2016).

### Patients With Abnormal Heart Rate Diurnal Pattern Have Higher Cardiovascular Disease Risk

**FIGURE 4:**
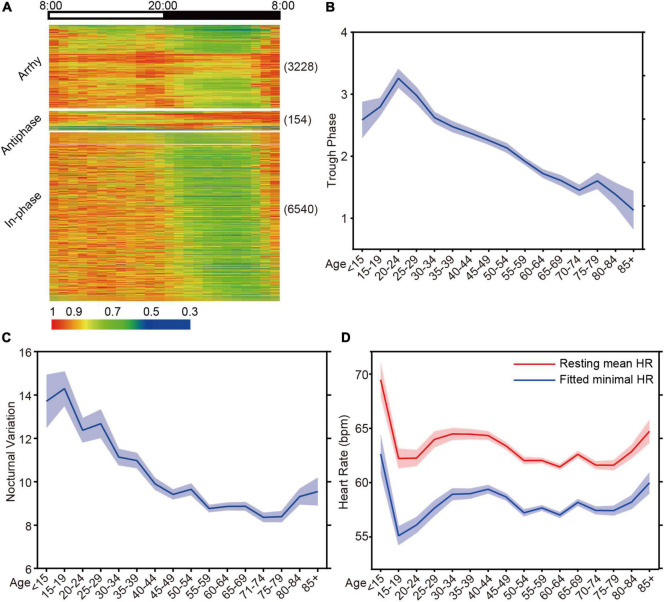
HR diurnal parameters are identified from Holter. **(A)** Cluster of the HR data from Holter patients based on the statistical *p*-value from JTK. Antiphase: *p* ≤ 0.05, N/D ≥ 1; Arrhy: arrhythmic, *p* > 0.05; Rhy: rhythmic, *p* ≤ 0.05, N/D < 1. Numbers in parentheses are the *N* for each group. **(B)** The mean trough phase of HR decreases with increasing age (*N* = 10094). **(C)** The mean nocturnal variation in HR shows similar age-dependent decreases (*N* = 10094). **(D)** The resting mean HR and fitted minimal HR decreases with increasing age (*N* = 10094).

To investigate the ability of HR diurnal patterns to facilitate disease risk prediction, we collected 10,094 Holter HR data sets from clinical patients. After excluding patients with artificial pacemakers, 9922 data sets were used to analyze. The age of the patients ranged from 8 to 97 years old, with a majority of the population (25 - 75% percentile) between 48 and 68 years of age (Supplementary Figure 3). We exported the cardiovascular pathological indicators from the Holter diagnostics and classified them into seven categories with 13 groups as CVD indices (Supplementary Table 1). Based on the rhythmicity analysis of JTK_CYCLE, the diurnal patterns of Holter patients were divided into arrhythmic (3,228 out of 9,922 patients, 32.5%) and rhythmic (6,694 out of 9,922 patients, 67.5%) clusters (Figure 4A). The rhythmic group was further clustered into the antiphase group (nighttime average HR/daytime average HR ≥ 1) and in-phase group (Figure 4A). We found that the arrhythmic and antiphase groups had higher risks of the indicated CVD indices than the in-phase rhythmic group by the Bonferroni test (Table 1).

**TABLE 1:** Confidence interval analysis (Bonferroni test) of the possibility of disease occurrence in different clusters.

Category	Rhythmic (*n* = 6540)	Arrhythmic (*n* = 3228)	Antiphase (*n* = 154)	Rhy vs. Arr		Rhy vs Anti	
	Mean (95% CI)	Mean (95% CI)	Mean (95% CI)	Diff	*P*	Diff	*P*
Atrial Events (yes or no)	0.19 (0.18, 0.20)	0.24 (0.23, 0.26)	0.48 (0.4,0.6)	0.05	< 0.001	0.29	< 0.001
Ventricular Events (yes or no)	0.25 (0.23, 0.26)	0.26 (0.24, 0.27)	0.36 (0.29, 0.44)	0.01	0.87	0.12	0.0024
Sinus Tachycardia (yes or no)	0.006 (0.004, 0.007)	0.008 (0.005, 0.01)	0.039 (0.008, 0.07)	0.002	0.72	0.033	< 0.001
Sinus Bradycardia (yes or no)	0.12 (0.11, 0.13)	0.16 (0.15, 0.18)	0.11 (0.06, 0.16)	0.04	< 0.001	−0.01	0.99
Conduction Block (yes or no)	0.11 (0.11, 0.12)	0.13 (0.12, 0.14)	0.18 (0.12, 0.24)	0.017	0.055	0.07	0.03
QRS (yes or no)	0.02 (0.02, 0.03)	0.04 (0.03, 0.05)	0.12 (0.07, 0.18)	0.02	< 0.001	0.10	< 0.001

*QRS: Q, R, and S waves.*

To further dissect the HR diurnal parameters to facilitate disease risk prediction, we employed the in-house algorithm to identify trough phase and nocturnal variation during resting time. When analyzing 5-year age intervals, age correlated strongly with HR trough phase (Figure 4B) and nocturnal variations (Figure 4C). Interestingly, the decrease in nocturnal HR variation decelerated after age 50 and rebounded after age 80, although the mechanisms are undetermined (Figure 4C). We noticed that both the resting mean HR and the fitted minimal HR rebounded after age 80, which may be the reason for the elevated nocturnal HR variation (Figure 4D).

### Extreme HR Diurnal Patterns Correlate With Cardiovascular Disease Indices

**FIGURE 5:**
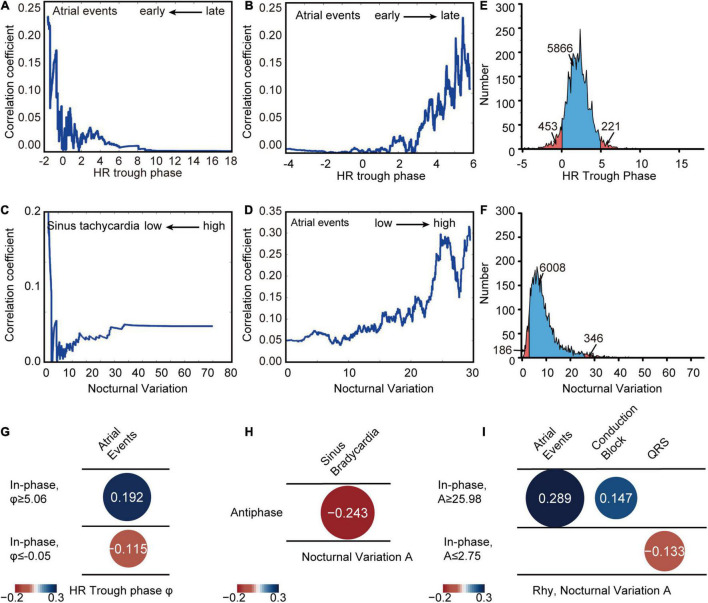
HR diurnal parameters correlate with CVD indices. **(A)** Partial correlation analysis between HR trough phase and atrial events in different parameter ranges earlier than different HR trough phase critical points (*x* axis). **(B)** Partial correlation analysis between HR trough phase and atrial events in different parameter ranges later than different HR trough phase critical points (*x* axis). **(C)** Partial correlation analysis between nocturnal variation and sinus tachycardia in different parameter ranges lower than different nocturnal variation critical points (*x* axis). **(D)** Partial correlation analysis between nocturnal variation and atrial events in different parameter ranges higher than different nocturnal variation critical points (*x* axis). **(E)** Distribution of HR trough phase values of Holter patients (blue: HR trough phase between –0.05 and 5.06) with the number of patients in each group labeled. **(F)** Distribution of nocturnal variation in Holter patients (blue: nocturnal variation between 2.75 and 25.98) with the number of patients in each group labeled. **(G)** Correlations between HR trough phase and CVD indices in the in-phase population with phases greater than 5.06 (Rhy, φ ≥ 5.06) (*N* = 221) and smaller than –0.05 (Rhy, φ ≤ –0.05) (*N* = 453) were calculated by spearman rank correlation. **(H)** Correlations between HR diurnal parameters (nocturnal variation) and CVD indices in the antiphase population (Antiphase) were calculated by spearman rank correlation (*N* = 154). **(I)** Correlation between nocturnal variation and CVD indices in the in-phase population with nocturnal variation, A, greater than 25.98 (Rhy, A ≥ 25.98) (*N* = 184) and lower than 2.75 (Rhy, A ≤ 2.75) (*N* = 346) were calculated by spearman rank correlation.

To determine the correlation between HR diurnal parameters and CVD indices, age- and sex-adjusted stepwise partial correlation analyses were performed to determine the critical point of HR trough phase and nocturnal variation in the rhythmic group. We found that patients with an HR trough phase between −0.05 and 5.06 (5,866 out of 6,540 patients, 89.7% in the in-phase group) and nocturnal variation between 2.75 and 25.98 (6,008 out of 6,540 patients, 91.9% in the in-phase group) had low risk of CVD indices by Spearman rank correlation (Figures 5A-D and Supplementary Figures 4, 5). Thus, the normal ranges with low risk of CVD indices were defined according to the above critical points (Figures 5E,F). Then, age- and sex- adjusted spearman rank correlation was used to further investigate the associations between various CVD indices and trough phase or nocturnal variation in outlier rank deviating from the critical points. HR trough phase (φ ≤ −0.05) or (φ ≥ 5.06) had a greatly increased association with atrial events (Figure 5G). Taken together, these data suggested that extreme HR diurnal patterns were significantly associated with cardiovascular outcomes. In the antiphase group, the nocturnal variation in HR showed the strongest correlation with sinus bradycardia (Figure 5H). Nocturnal variation (A ≥ 25.98) was significantly associated with atrial events and conduction block (Figure 2I). When nocturnal variation was lower than 2.75, it showed a clear correlation with QRS (Figure 5I). Further analysis of antiphase patients indicated that the use of BETALOC or other cardiac-related drugs did not change the HR phase, nocturnal variation, or the night/day HR ratio (Supplementary Figure 6).

### Heart Rate Diurnal Parameters From Wrist Wearables Are Nearly Identical to Those From Holter Monitors

**FIGURE 6:**
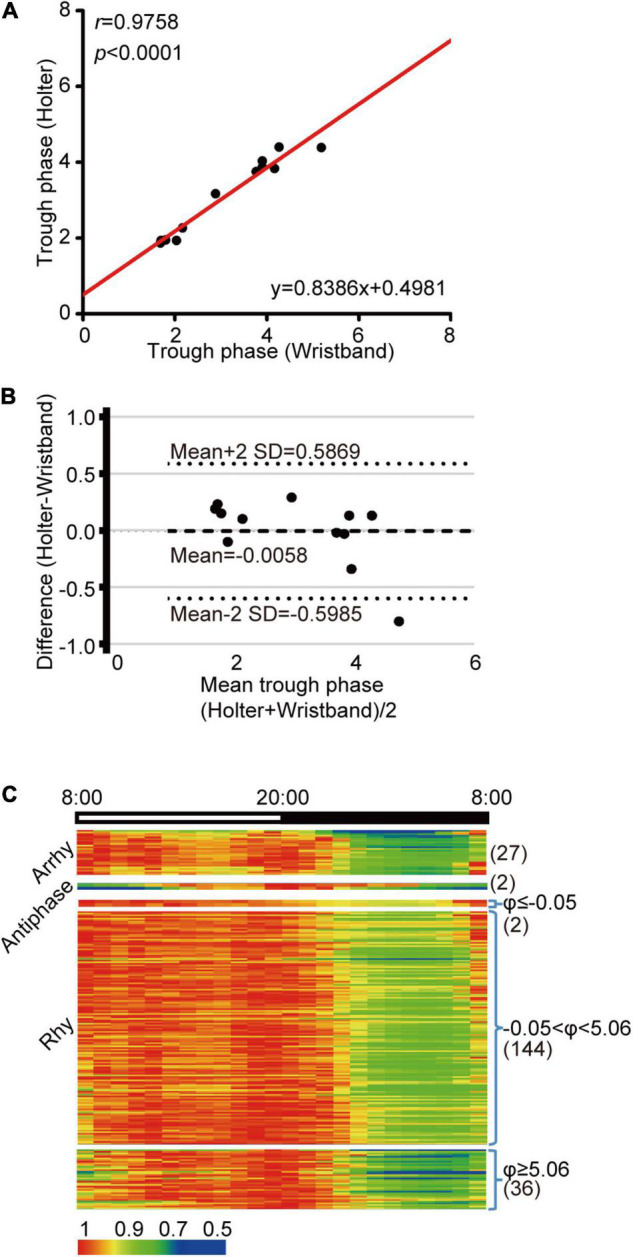
HR diurnal parameters from wrist wearables accurately reflect heart diurnal patterns. **(A)** Linear regression of trough phase values derived from wristband and Holter HR monitoring on the same day. Black dots indicate data from each individual, and the red line represents the linear regression curve. *N* = 12, *r* = 0.9758, *p* < 0.0001. **(B)** Bland-Altman plot for comparison of trough phase from wristband and Holter, with the representation of mean biases (solid line) and the limits of agreement (dotted lines), from Mean-2SD to Mean + 2SD. **(C)** Cluster of the HR data from wristband volunteers based on the JTK value and the mean night-to-day HR ratio (*N* = 211). Numbers in parentheses are the N for each group.

A Holter monitor is a precise medical grade device, but it is inconvenient for daily HR monitoring. Currently, wrist wearables are becoming popular and are easy to be used as HR recording. We wondered whether HR parameters obtained from wrist wearables are close to that from Holter. First, we sought to assess the accuracy of wristband-based HR data relative to Holter monitoring. We recruited 12 volunteers who were equipped with both a Holter monitor and wristband on the same day and compared the device performances by linear correlation of trough phase results. We found that the trough phases estimated from these two devices were well correlated (Figure 6A, *r* = 0.9758, *p* < 0.0001, Supplementary Figure 7). Bland-Altman plots demonstrated that the 95% limits of agreement were within −0.0058 ± 0.06 h (Figure 6B), indicating that wristband-based HR tracking is feasible for HR diurnal pattern determination.

We found that 182 individuals showed rhythmicity (182 out of 211 participants, 86.3%), and 27 individuals showed arrhythmicity (27 out of 211 participants, 12.8%) (Figure 6C), which was lower than the 32.5% in the Holter patient population. In addition, two volunteers were clustered into the antiphase pattern (2 out of 211 participants, 1%), which was also lower than that in Holter patients (2.3%). When we further queried the two antiphase participants, one reported temporary sleep deprivation, while the other complained of tachycardia, implying a potential risk of heart function.

## Discussion

In this study, we developed a comprehensive analytical strategy to classify diurnal patterns and extract HR diurnal parameters for the cardiovascular system. We also validated that this analytical strategy can be applied to long-term HR data collected from wearable devices (wristbands) to depict the longitudinal trajectories of personalized circadian function. Given the growing popularity of wearable health care devices, the classification and analysis of HR data will be helpful.

When comparing our results with other circadian measurements, we noticed deviations between HR trough phase and DLMO, which suggests that different circadian output markers have distinct features despite the overall correlation. The HR diurnal parameters directly reflect the dynamic change in cardiac function in day-night cycles. Therefore, our strategy could provide a better reference to guide time-sensitive cardiac treatment but is limited to HR diurnal parameters. We didn’t validate the HR diurnal parameters against results generated from skin or blood samples. Further comparative studies may be needed to dissect the timing differences between various methods.

Under limited conditions, we studied the simplified relationship between HR diurnal parameters and CVD risks. We identified two population clusters, antiphase and arrhythmic groups, associated with CVD indices. The major finding was that extreme heart diurnal patterns (extremely advanced, extremely late, and antiphase) correlated with a higher risk of CVD, especially atrial events such as atrial fibrillation/flutter, atrial load, and atrial tachycardia. These results persisted after adjustment for age and sex, which is consistent with the increased disease occurrence among shift workers (Knutsson et al., 1986). We thus suggest that misalignment between the central clock, the cardiovascular clock, and environmental cues may play an important role in heart physiology and pathology. This enables us to consider heart diurnal patterns to be a modifiable risk factor with an available preventive target. Extreme heart diurnal patterns are attributes of cardiovascular diseases and can potentially be used to guide intervention. Circadian biology is becoming a critical element in precision and personalized medicine (Cederroth et al., 2019; Ruben et al., 2019; Ju and Zhang, 2020; Wu et al., 2020; López-Otín and Kroemer, 2021). Progress in this field has encouraged efficient and reliable methods that are inexpensive and non-invasive for predicting circadian rhythm. Our method and results provide a roadmap for future direction. Extreme HR phase and nocturnal variation are highly associated with specific CVDs. Wrist wearables can generate comparable HR diurnal parameters for CVD risk prediction.

## Data Availability Statement

The raw data supporting the conclusions of this article will be made available by the authors, without undue reservation.

## Ethics Statement

The studies involving human participants were reviewed and approved by Ethics Committee of Soochow University. The patients/participants provided their written informed consent to participate in this study.

## Author Contributions

YX and FZ are the guarantors of this work. LY, ZY, WZ, and TZ developed the algorithms to determine the HR diurnal parameters. YG and TZ performed the volunteer data collection and statistical analysis with insights from HT. YD recruited the volunteers, collected the volunteer samples and performed the ELISA. ZL constructed the local database. XD performed the clinical data collection with technical help from XH, CZou, YZ, GW, and JH provided comments and technical support. FZ drafted the manuscript with comments from all authors. YX edited the manuscript. All authors read and approved the final manuscript.

## Conflict of Interest

The authors declare that the research was conducted in the absence of any commercial or financial relationships that could be construed as a potential conflict of interest.

## Publisher’s Note

All claims expressed in this article are solely those of the authors and do not necessarily represent those of their affiliated organizations, or those of the publisher, the editors and the reviewers. Any product that may be evaluated in this article, or claim that may be made by its manufacturer, is not guaranteed or endorsed by the publisher.
