# Supplementary material for: Analysis of Diurnal Variations in Heart Rate: Potential Applications for Chronobiology and Cardiovascular Medicine
Source: Front Physiol. 2022 Mar 8;13:835198. doi: 10.3389/fphys.2022.835198 (PMC8958024; doi:10.3389/fphys.2022.835198)
Supplement: Supplementary file 1 [file Data_Sheet_1.docx]

**The Key analytical algorithm for HR data**

**Determination of HR trough phase, slopes, and variation**

***1. Preprocessing***

Considering the differences in the timestamp storage format of different software, the original data need to be aligned before analysis. The datasets are also screened for integrity and general pattern to exclude poor datasets as a result of abnormal device wearing. Data are divided by day from 2 p.m. through 2 p.m. the following day.

***2. Smoothing filter***

The original HR curve is derived from preprocessed data, *y*, which under most circumstances consists of a macroscopic circadian curve (low frequency) and multiple local oscillations (high frequency). To investigate the circadian curve of HR in the sleep stage, high-frequency noise needs to be filtered out by a low-frequency filter that greatly attenuates the high-frequency components of the curve. In our algorithm, a Butterworth low-pass filter is used.

***3. Night window***

Instead of defining a fixed day/night window, the duration of the night period for obtaining the nighttime parameter is searched and determined using a sliding window.

3.1) Using smooth filtered data, the critical HR value, B_inf, is defined as the smaller value between the mean and the median of the HR data throughout a day.

3.2) A default time period is defined as an interval of 240 (min) (1). CountPoint, another parameter for determining the night period, is the number of data points below B_inf in the time window of [1: interval (1)].

3.3) Classification

If CountPoint = 0, delay the time window (slide rightwards) for a length of interval (1) and repeat step 3.2;

If 0 < CountPoint < interval (1), slide the time window 1 unit rightwards and repeat step 3.2;

When CountPoint = 240, the first time point of the time window is defined as the start point of the night window, *t_s_*.

3.4) Define the end point of the night window, *t_e_*, using a similar strategy as in steps 3.2 & 3.3 but by advancing the time window.

3.5) The interval (1) can be changed to 120 (min) if no results are obtained with the default setting for short sleepers.

3.6) If the number if increasing data points is more than twice the number decreasing data points, *t_s_* can be advanced *u_s_* hours, where 0<= *u_s_* <1.

3.7) If the number of decreasing data points is more than twice the number increasing data points, *t_e_* can be advanced *u_e_* hours, where 0<= *u_e_* <1.

3.8) The sleep period is between *t_s_* - *u_s_* and *t_e_* + *u_e_*, and the duration is *Ds* = (*t_e_* + *u_e_*) - (*t_s_* - *u_s_*).

3.9) If the sleep period data meet the following criteria, the data on this day will not be analyzed.

1. *Ds* is longer than 15 hours.

2. Missing data are continuous and form a gap longer than 3 hours.

3. There are less than 6 hours, where the number of data points is greater than 10 within each hour.

4. The range of data within 4 hours is less than 6.

***4. Slopes and other parameters***

4.1) The sleep period is divided into three equal parts, *P_1_, P_2_* and *P_3_*. *P_1_* is [*t_s_*- *u_s,_ t_s_*- *u_s_*+ *Ds/3*]*, P_2_* is [*t_s_*- *u_s_*+ *Ds/3, t_s_*- *u_s_*+ *2Ds/3*], and *P_3_* is [*t_s_*- *u_s_*+ *2Ds/3, t_e_*+*u_e_*].

4.2) The decreasing index *K_de_* is used to determine the time window to sleep, which can be described as *K_de_*=0.3*K_den_*+0.7*K_dev_*, where *K_den_* is the number of decreasing data points within a 50-minute window, and *K_dev_* is the sum of the drops in the decreasing data points within 50 minutes. *K_den_* and *K_dev_* can be described as

$K_{den}=\sum_{i=1}^{n} k_{deni}$,

$k_{deni}=\left\{ \begin{matrix} 1 & y_{di}<0 \\ 0 & y_{di}\geq0 \end{matrix} \right.$ ,

$K_{dev}=\sum_{i=1}^{n} k_{devi}$,

$k_{deni}=\left\{ \begin{matrix} -y_{di} & y_{di}<0 \\ 0 & y_{di}\geq0 \end{matrix} \right.$,

$y_{di}=y_{i+1}-y_{i}$,

where *y_i_* is the *i*-th HR data point within the 50-minute window.

4.3) The *K_de_* of each 50-minute window in *P_1_* is calculated. The 50-minute window corresponding to the maximal *K_de_* in this step is denoted *P_s_*.

4.4) The beginning time of the time window to sleep, *t_bs_*, is the time of the maximum HR within the first half of *P_s_*. The end time of the time window to sleep, *t_es_*, is the time of the minimum HR within the second half of *P_s_*.

4.5) The HR decreasing rate of *P_s_*, *R_drs_*, is defined as $R_{drs}=\frac{K_{devs}}{50}$, where *K_dvs_* is the sum of the drops in the decreasing data points in *P_s_*.

4.6) The increasing index *K_in_* is used to determine the time window to sleep, which can be described as *K_in_*=0.3*K_inn_*+0.7*K_inv_*, where *K_inn_* is the number of decreasing data points within a 50-minute window, and *K_inv_* is the sum of the drops in the decreasing data points within 50 minutes. *K_inn_* and *K_inv_* can be described as

$K_{inn}=\sum_{i=1}^{n} k_{inni}$,

$k_{inni}=\left\{ \begin{matrix} 1 & y_{di}>0 \\ 0 & y_{di}\leq0 \end{matrix} \right.$ ,

$K_{inv}=\sum_{i=1}^{n} k_{invi}$,

$k_{inni}=\left\{ \begin{matrix} y_{di} & y_{di}>0 \\ 0 & y_{di}\leq0 \end{matrix} \right.$,

$y_{di}=y_{i+1}-y_{i}$,

where *y_i_* is the *i*-th HR data point within the 50-minute window.

4.7) The *K_in_* of each 50-minute window in *P_3_* is calculated. The 50-minute window corresponding to the maximal *K_in_* in this step is denoted *P_as_*.

4.8) The beginning time of the time window to sleep, *t_bas_*, is the time of the minimum HR within the first half of *P_as_*. The end time of the time window to sleep, *t_eas_*, is the time of the maximum HR within the second half of *P_as_*.

4.9) The HR increasing rate of *P_as_* is *R_inras_*, defined as $R_{inras}=\frac{K_{invas}}{50}$, where *K_invas_* is the sum of the drops in the decreasing data points in *P_as_*.

***5. Filtering out high-frequency peaks***

HR is mainly regulated by the sleep cycle (high frequency) and the circadian rhythm (low frequencies) during the night. Fluctuations in HR data obtained from step 3) should be filtered to remove the influence of sleep cycle on HR.

5.1) Step 5.1) and 5.2) are for peaks with widths of approximately 3 minutes. The threshold, *r_p_*, is the lower quartile of HR on that day. The other two parameters, time duration, *t_interval_*, and peak width, *w*, are 3 and 0, respectively.

5.2) HR data corresponding to positions of fluctuations defined in step 6 based on the three parameters outlined in step 5.1) are removed.

5.3) Steps 5.3) and 5.4) are performed to remove peaks with widths of approximately 10 to 15 minutes following step 5.2.

5.4) HR data from step 3) are binned at 5-minute intervals, and step 6 is performed with the following parameters to obtain positions of fluctuations. *t_interval_* is 11, *w* is 0, and *r_p_* is twice that of step 5.1).

5.5) Steps 5.5) and 5.6) are performed to remove peaks with widths of approximately 60 minutes following step 5.4.

5.6) HR data from step 3) are binned at 5-minute intervals, and step 6) is performed with the following parameters to obtain positions of fluctuations. *t_interval_* is 60, *w* is 1, and r_p_ is 10.

***6. Positions of fluctuations to be removed***

Step 6) is included as part of step 5); *y_di_* is the first-order difference of HR at time *t_i_*, and *y_di_* can be described as

$$y_{di}=y_{i+1}-y_{i}$$

where *y_i_* is the *i*-th HR data in a time window of the sleep period, which is denoted *P_i_*. The time window duration, *t_interval_*, is determined in step 5. The initial time window in this step is [*t_s_*-*u_s,_ t_s_*-*u_s+_ t_interval_*].

6.1) If *w* is 0, then

6.1.1) If *y_maxi_*-*y_mini_*< *r_p_*, the process moves to step 6.1.4), where *y_maxi_* is the maximum HR in *P_i_* , and ymini is the minimum HR in *P_i_*. If *y_maxi_*-*y_mini_*≥r_p_, *P_i_* is divided into three equal parts, *P_i1,_ P_i2_* and *P_i3_,* as step 4.1. *S_i1_*, *S_i2_* and *S_i3_* are the sums of HR in *P_i1,_ P_i2_* and *P_i3_*, respectively.

6.1.2) If *S_i2_*> *S_i1_* and *S_i2_*> *S_i3_*, the process moves to step 6.1.3. Otherwise, the process moves to step 6.1.4).

6.1.3) *t_h_* is the time of the first time point when *y_di_* is greater than zero. If *t_h_* does not exist, the process moves to step 6.1.4). *t_t_* is the time of the last time point when *y_di_* is less than zero. The HR data between *t_h_* and *t_t_* should be removed. If *t_t_* does not exist, the process moves to step 6.1.4).

6.1.4) The time window is delayed by one time point.

6.2) If *w* is 1, then

6.2.1) If *y_maxi_*-*y_mini_*< *r_p_*, the process moves to step 6.2.4), where *y_maxi_* is the maximum HR in *P_i_*, and ymini is the minimum HR in *P_i_*. If *y_maxi_*-*y_mini_*≥*r_p_*, *P_i_* is divided into three equal parts, *P_i1,_ P_i2_* and *P_i3_* as in step 4.1. *S_i1_*, *S_i2_* and *S_i3_* are the sums of HR in *P_i1,_ P_i2_* and *P_i3_*, respectively.

6.2.2) If *S_i2_*> *S_i1_* and *S_i2_*> *S_i3_*, the process moves to step 6.2.3. Otherwise, the process moves to step 6.2.4).

6.2.3) HR data meeting the following criteria should be removed.

*y_i_*>*y_maxi2_*- *r_p_*

6.2.4) The time window is delayed by one time point.

***7. Estimating trough phase and HR variation***

7.1) Preprocessed HR data from step 5 are fitted by cosine functions via MATLAB’s lsqcurvefit function.

7.2) Trough phases are the times of the lowest points on the fitted curves in 7.1). *A_mpn_* is the nocturnal HR variation, and *A_mpd_* is the diurnal HR variation. *A_mpn_* and *A_mpd_* can be described as

*A_mpn_*=2(*E_n_*-*y_minc_*)

*A_mpd_*=2(*E_d_*-*y_minc_*)

where *E_n_* and *E_d_* are the mean of nighttime and full-day HRs. *y_minc_* is the minimum HR on the fitted curve in 7.1).

***8. Estimating local peaks and troughs***

8.1) the Time window *Q_i_* duration, *t_interval_*, in this study is 50 minutes, and the initial time window in this step is [*t_s_*-*u_s,_ t_s_*-*u_s+_t_interval_*]. *Q_i_* is divided into three equal parts, *Q_i1,_ Q_i2_* and *Q_i3_* according to step 4.1. *y_maxi_* and *y_mini_* are the maximum and minimum HRs in *Q_i_*. *t_maxi_* and *t_mini_* are the time of maximum and minimum HRs in *Q_i_*. The threshold *r_q_* is 2.

8.2) If *y_maxi_*- *y_mini_*> *r_q_* and *t_maxi_* are within *Q_i2_*, the process moves to step 8.3. Otherwise, the process moves to step 8.4).

8.3) If *Q_i_* meets the following criteria, *y_maxi_* is the local peak value.

*E_q2_*>*E_q1_*

*E_q2_*>*E_q3_*

*y_maxi_*- *y_mini1_*> *r_q_*

*y_maxi_*- *y_mini3_*> *r_q_*

where *E_q1_*, *E_q2_* and *E_q3_* are the mean HRs in *Q_i1,_ Q_i2_* and *Q_i3_,* respectively. *y_mini1_* and *y_mini3_* are the minimum HRs in *Q_i1_* and *Q_i3_*.

8.4) If *y_maxi_*- *y_mini_*> *r_q_* and *t_mini_* are within *Q_i2_*, the process moves to step 8.5. Otherwise, the process moves to step 8.6).

8.5) If *Q_i_* meets the following criteria, *y_mini_* is the local trough value.

*E_q2_*<*E_q1_*

*E_q2_*<*E_q3_*

*y_maxi_*- *y_mini1_*< *r_q_*

*y_maxi_*- *y_mini3_*<*r_q_*

8.6) The time window is delayed by one time point.

8.7) If there is no trough between two peaks, the minimum HR between the two peaks is identified as the trough. If there is no peak between the two troughs, the maximum HR between the two troughs is identified as the peak.


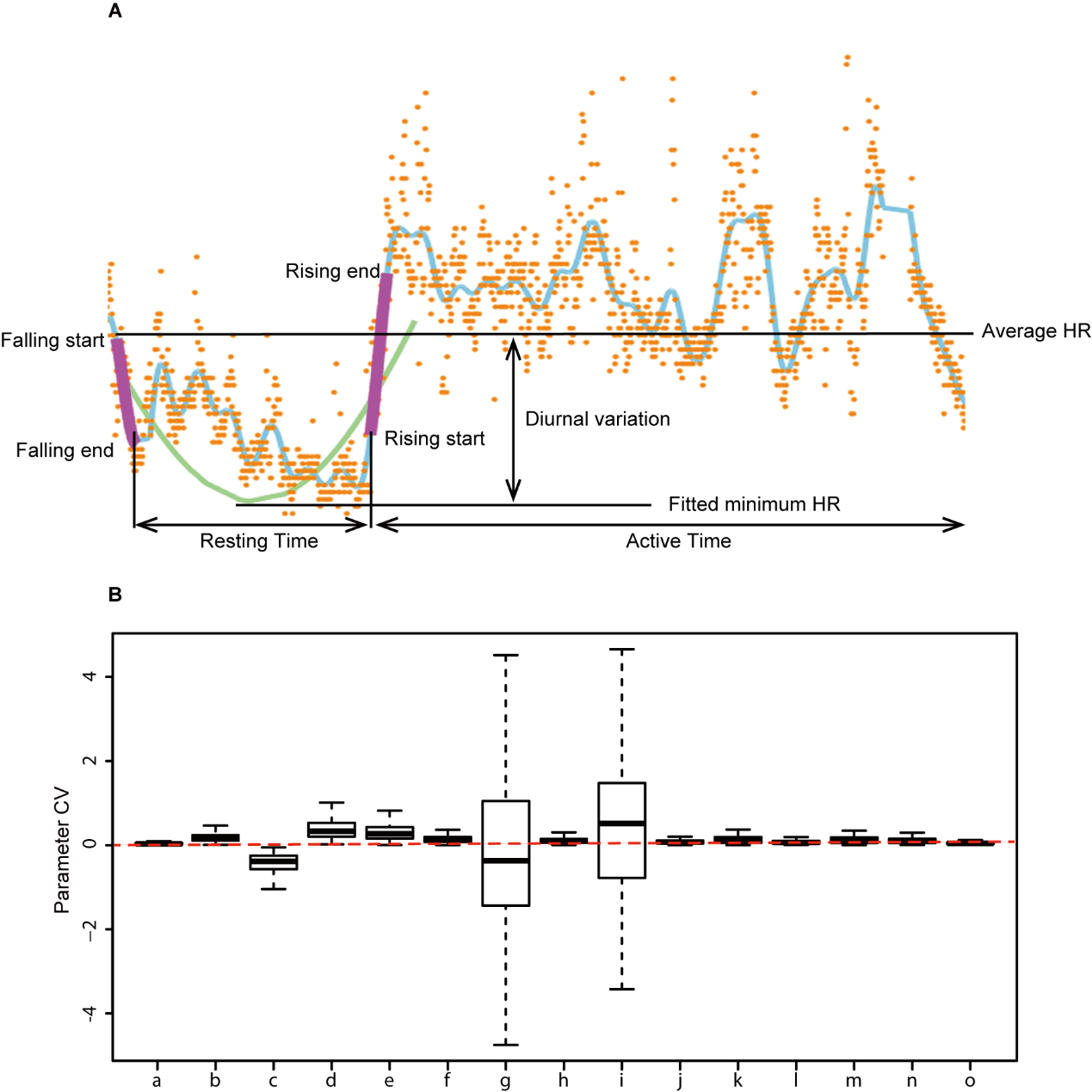


**Supplementary Figure 1.** (A) Heart rate diurnal parameters calculated from the in-house developed algorithm. Yellow dots: the raw data, green curve: fitting curve, purple lines: the falling and rising curve.Diurnal variation is the difference between fitted minimum HR and whole-day mean HR. (B) Interday variation of HR diurnal parameters. The interday coefficient of variation (CV) of all HR diurnal parameters was analyzed using data of the first 3 workdays retrieved from all 211 volunteers. The box plot shows the distribution of SD values for each parameter. a: resting mean HR, b: diurnal variation, c:falling slope, d:rising slope, e:resting SD, f: whole-day SD, g: falling start time, h: falling start HR, i: falling end time, j:falling end HR, k:rising start time, l:rising start HR, m:rising end time, n:rising end HR, o:fitted minimum HR.


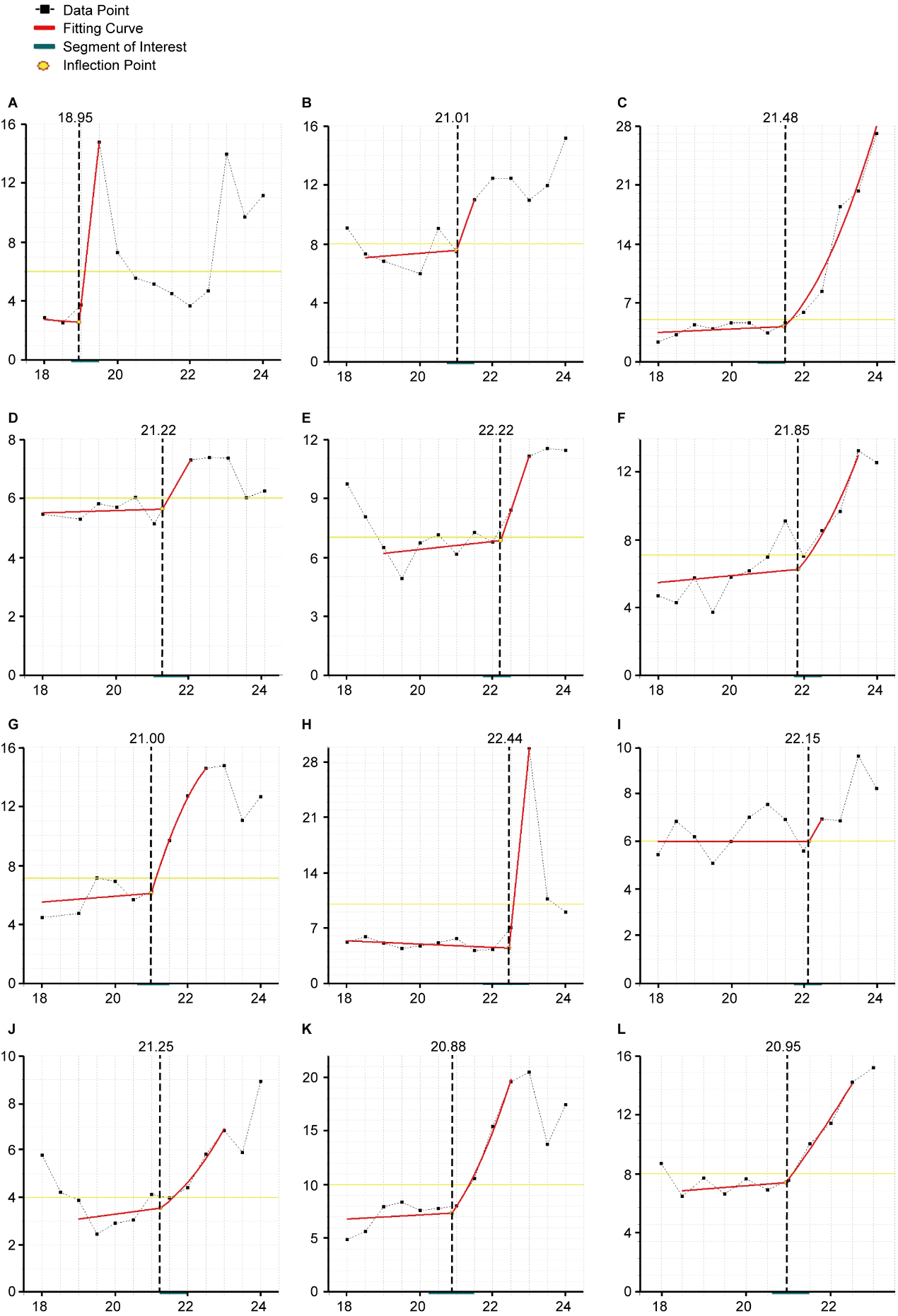


**Supplementary Figure 2.** Dim-light melatonin onset (DLMO) results of 12 volunteers. Twelve volunteers were recruited for DLMO sample collection. Saliva samples were collected every 30 minutes during 18:00-24:00 in a lightproof room. Melatonin concentrations in saliva samples were detected using a commercial ELISA kit, and the onset time points (inflection point) were analyzed using the ‘hockey stick’ method as shown in the figure.


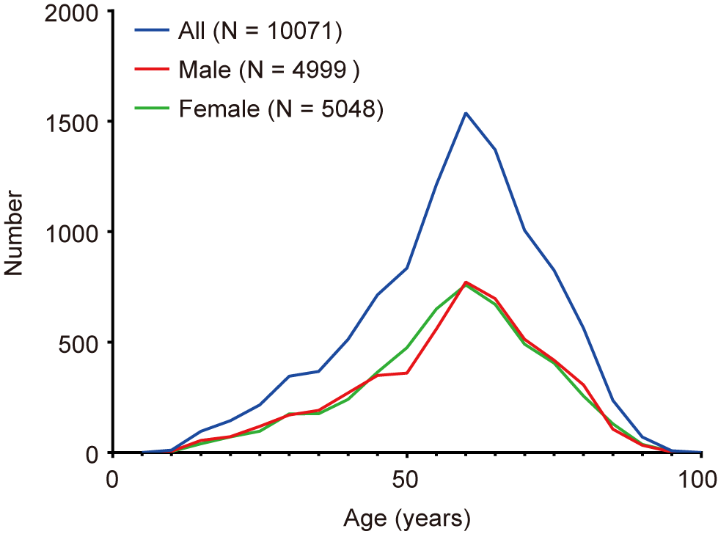


**Supplementary Figure 3.** Age of Holter patients. Age distribution of 10,071 Holter patients. 10,095 Holter patients were subjected to analyses, among which 10,071 had age info and 10,047 had gender info. The number of male and female patients and their age distribution are similar.


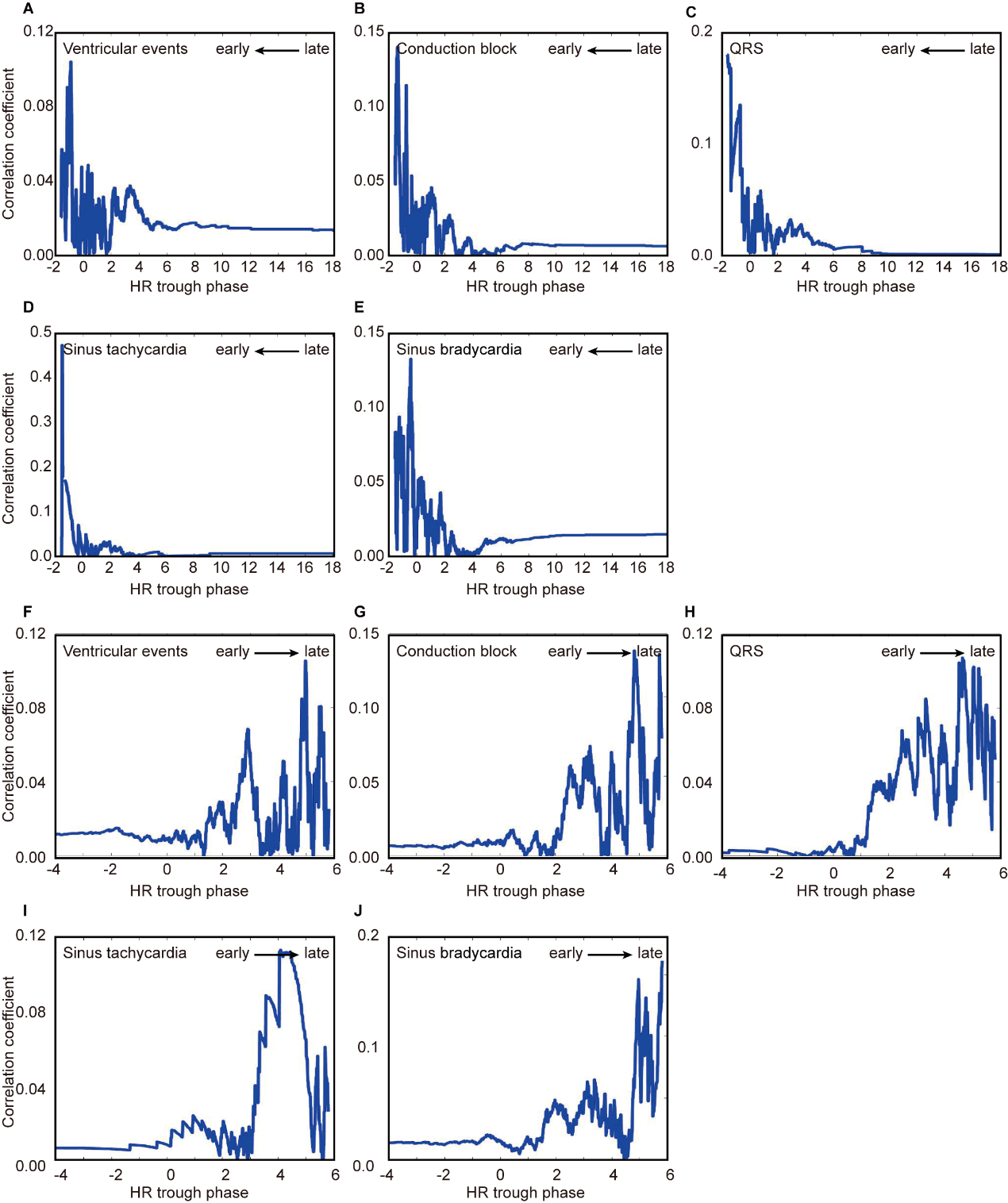


**Supplementary Figure 4.** Determination of Critical points for HR trough phase by Spearman rank correlation. A-E, Partial correlation analysis between HR trough phase and ventricular events (A), conduction block (B), QRS (C), sinus tachycardia (D), sinus bradycardia (E) in different parameter ranges before a certain HR trough phases (x axis). F-J, Partial correlation analysis between HR trough phase and ventricular events (F), conduction block (G), QRS (H), sinus tachycardia (I), sinus bradycardia (J) in different parameter ranges after a certain HR trough phases (x axis).


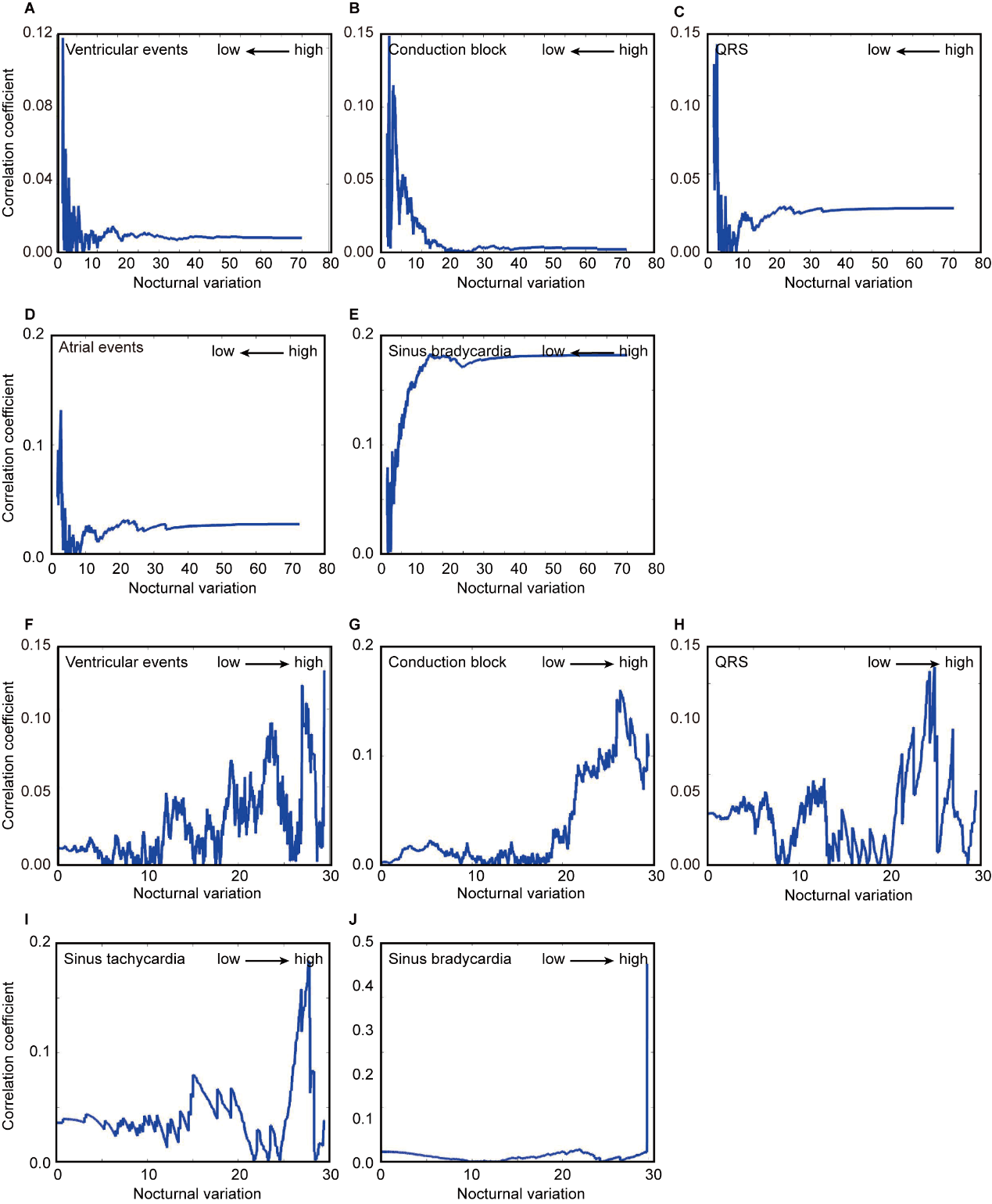


**Supplementary Figure 5.** Determination of Critical points for nocturnal HR variation by Spearman rank correlation. A-E, Partial correlation analysis between nocturnal variation and atrial ventricular events (A), conduction block (B), QRS (C), atrial events (D), sinus bradycardia (E) in different parameter ranges less than a certain nocturnal variation (x axis). F-J, Partial correlation analysis between nocturnal variation and ventricular events (F), conduction block (G), QRS (H), sinus tachycardia (I), sinus bradycardia (J) in different parameter ranges greater than a certain nocturnal variation (x axis).


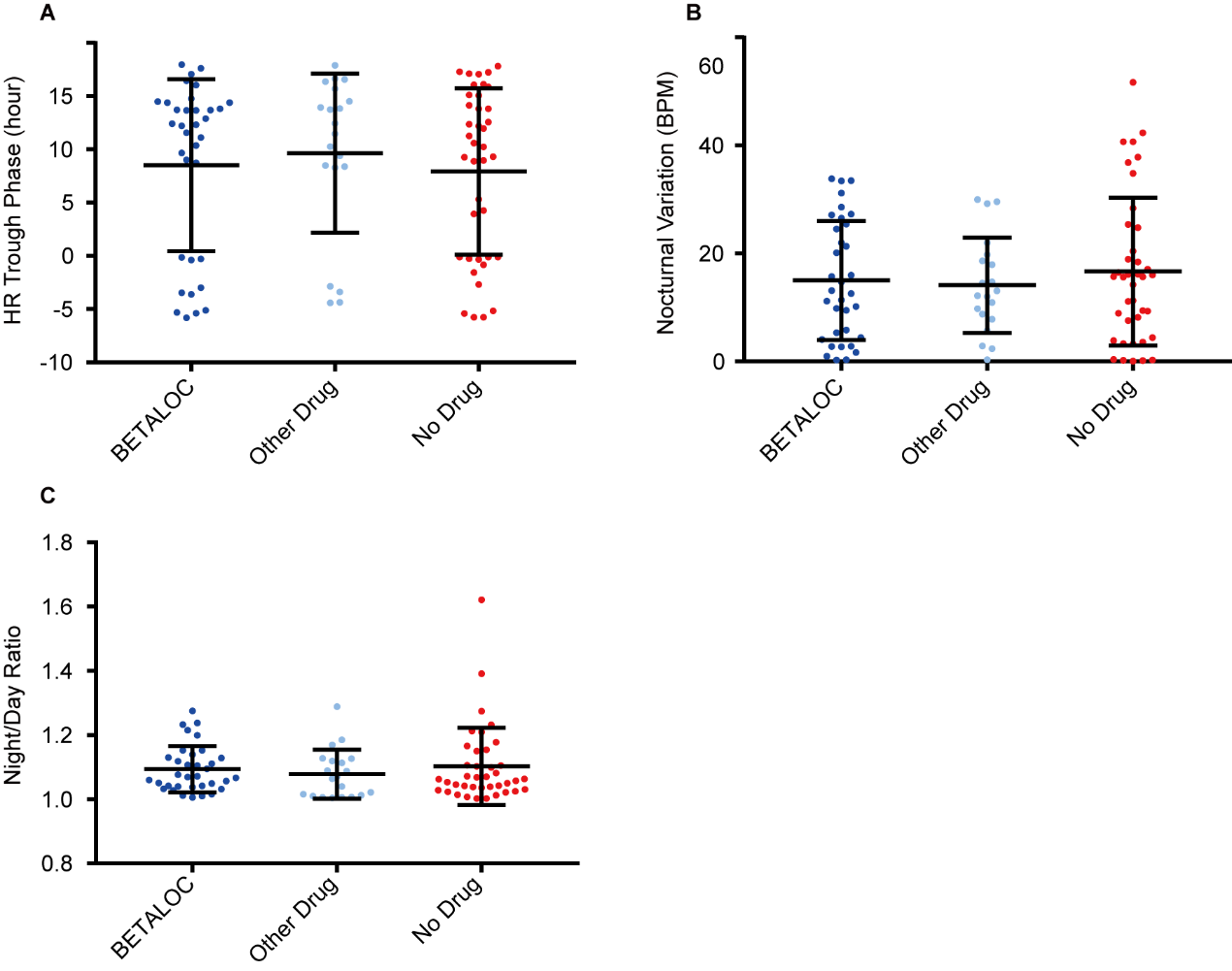


**Supplementary Figure 6.** Comparison of HR diurnal parameters of antiphase patients with different drug treatments. A-C, 93 antiphase patients (54 in cardiovascular wards and 39 in non-cardiovascular wards) were further divided into three groups according to their medication record: BETALOC – patients prescribed with BETALOC; Other Drug – patients prescribed with drugs other than BETALOC, such as digoxin, isoprenaline or amiodarone by intravenous infusion; No Drug: patients had no medication records of cardiovascular drugs. HR trough phase (A), nocturnal variation (B) and Night/Day ratio (C) of antiphase patients in different groups were compared. No significant differences were detected.


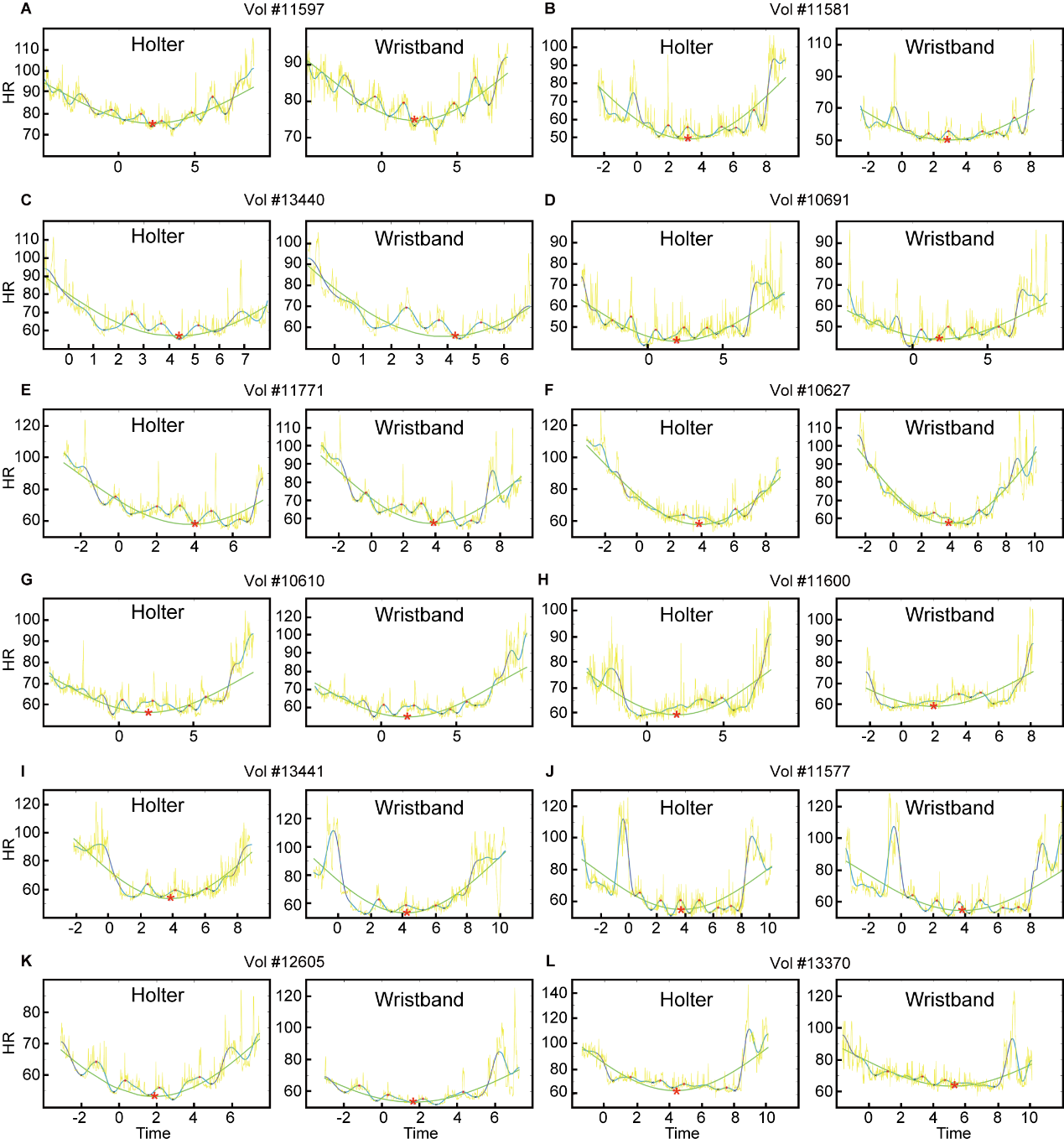


**Supplementary Figure 7.** Comparison of analytical results with HR data from Holter and wristband. A-L, Parallel comparison of HR curves from individual volunteer using HR data collected by Holter (left) and wristband (right). Red asterisk: fitted HR trough.

Supplementary Table 1. Cardiovascular Disease Indices Exported from the Holter Data

| **Category** | **Index** | **Keywords** | **Screening Keywords** |
| --- | --- | --- | --- |
| tachyarrhythmia | atrial | atrial fibrillation, atrial flutter | atrial fibrillation, atrial flutter |
|  | atrial overload | >10% atrial overload | atrial premature beat counts >10% |
|  | atrial tachycardia | atrial tachycardia | atrial tachycardia |
|  | ventricular | ventricular fibrillation, ventricular flutter | ventricular fibrillation, ventricular flutter |
|  | ventricular overload | >10% ventricular overload | ventricular premature beat counts >10% |
|  | ventricular tachycardia | ventricular tachycardia | ventricular tachycardia |
|  | sinus tachycardia | sinus tachycardia | sinus tachycardia |
| bradyarrhythmia | atrioventricular block | 2nd degree type 1 atrioventricular block | 2nd degree type 1 atrioventricular block |
|  |  | 2nd degree type 2 atrioventricular block | 2nd degree type 2 atrioventricular block |
|  |  | 3rd degree atrioventricular block | 3rd degree atrioventricular block |
|  | other conduction block | intraventricular block | intraventricular block |
|  |  | left bundle branch block | left bundle branch block |
|  |  | incomplete left bundle branch block |  |
|  |  | complete left bundle branch block |  |
|  |  | left anterior fascicular block | left anterior fascicular block |
|  |  | left posterior fascicular block | left posterior fascicular block |
|  |  | right bundle branch block | right bundle branch block |
|  |  | incomplete right bundle branch block |  |
|  |  | complete right bundle branch block |  |
|  | sinus bradycardia | sinus bradycardia | sinus bradycardia, mean HR <45 |
|  |  | sinoatrial block | sinoatrial block |
|  |  | sinus arrest | sinus arrest, longest RR >3s |
| pacemaker | pacemaker | pacemaker | pacemaker |

Supplementary Table 2 Analytical Parameters for Heart Diurnal Pattern

| **Parameters** | **Description** |
| --- | --- |
| trough phase | phase of the fitted trough of heart rate during resting |
| fitted minimum HR | minimum heart rate at the fitted trough |
| average resting HR | average heart rate during resting |
| nocturnal variation | oscillation range of HR during the night: the difference between resting average HR and the fitted minimum HR |
| diurnal variation | oscillation range of HR during a whole day: the difference between whole-day average HR and the fitted minimum HR |
| falling slope | The switch between sleeping and awakening is accompanied by falling or rising heart rate. During data regression and modeling, the curve of heart rate falling and rising can be drawn, and corresponding parameters are obtained, including slope, start/end time point and the corresponding heart rate. |
| falling start time |  |
| falling start HR |  |
| falling end time |  |
| falling end HR |  |
| rising slope |  |
| rising start time |  |
| rising start HR |  |
| rising end time |  |
| rising end HR |  |
| night/day ratio | mean daytime (8:00-20:00) HR/mean nighttime HR |
